# The Left Fusiform Gyrus is a Critical Region Contributing to the Core Behavioral Profile of Semantic Dementia

**DOI:** 10.3389/fnhum.2016.00215

**Published:** 2016-05-19

**Authors:** Junhua Ding, Keliang Chen, Yan Chen, Yuxing Fang, Qing Yang, Yingru Lv, Nan Lin, Yanchao Bi, Qihao Guo, Zaizhu Han

**Affiliations:** ^1^State Key Laboratory of Cognitive Neuroscience and Learning, IDG/McGovern Institute for Brain Research, Beijing Normal UniversityBeijing, China; ^2^Department of Neurology, Huashan Hospital, Fudan UniversityShanghai, China; ^3^Department of Radiology, Huashan Hospital, Fudan UniversityShanghai, China; ^4^Institute of Psychology, Chinese Academy of SciencesBeijing, China

**Keywords:** lesion-behavior mapping, fusiform gyrus, co-atrophy, semantic dementia, semantic deficits

## Abstract

Given that extensive cerebral regions are co-atrophic in semantic dementia (SD), it is not yet known which critical regions (SD-semantic-critical regions) are really responsible for the semantic deficits of SD. To identify the SD-semantic-critical regions, we explored the relationship between the degree of cerebral atrophy in the whole brain and the severity of semantic deficits in 19 individuals with SD. We found that the gray matter volumes (GMVs) of two regions [left fusiform gyrus (lFFG) and left parahippocampal gyrus (lPHG)] significantly correlated with the semantic scores of patients with SD. Importantly, the effects of the lFFG remained significant after controlling for the GMVs of the lPHG. Moreover, the effects of the region could not be accounted for by the total GMV, general cognitive ability, laterality of brain atrophy, or control task performance. We further observed that each atrophic portion of the lFFG along the anterior–posterior axis might dedicate to the loss of semantic functions in SD. These results reveal that the lFFG could be a critical region contributing to the semantic deficits of SD.

## Introduction

Semantic dementia (SD, which is generally referred to as semantic variant primary progressive aphasia) is characterized by the selective deterioration of semantic knowledge (Snowden et al., 1989; Hodges et al., 1992). The neuroanatomical profile of this disease includes progressive brain atrophy, with the earliest and most severe atrophy occurring in the temporal poles (Mesulam et al., 2012, 2013). These findings promote the assumption that the temporal pole is the critical cortical region responsible for the semantic impairments observed in SD patients (i.e., SD-semantic-critical region; Seeley et al., 2009; Guo et al., 2013). However, this assumption should be considered with caution given that there are many atrophic cortical regions in SD (i.e., SD-cortical-atrophic regions; Rohrer et al., 2009; La Joie et al., 2014), and some of these regions have been correlated with the severity of semantic deficits in SD (i.e., SD-semantic-correlated regions; Galton et al., 2001; Rosen et al., 2002; Adlam et al., 2006). Therefore, a potential limitation of these studies is the lack of identification of the SD-semantic-critical regions from these SD-semantic-correlated regions because all the SD-semantic-correlated regions are atrophic (e.g., Good et al., 2002; Ogar et al., 2011). Therefore, the results attributed to a given SD-semantic-correlated region might actually arise from the atrophy of another region. Thus, an SD-semantic-critical region should maintain a significant association with semantic performance in SD after the influence of the other SD-semantic-correlated regions is adjusted.

Mion et al. (2010) conducted an elegant functional positron emission tomography (PET) study in which they found that the fusiform gyrus (FFG), but not the temporal pole, exerted a significant effect on semantic disruptions in SD by performing regression analyses that simultaneously considered the effects of eight regions (the bilateral temporal poles, FFGs, superior temporal gyri, and inferior frontal gyri). However, it is important to explore whether structural changes in the FFG are critical for the semantic deficits of SD. Moreover, it is necessary to examine whether there are other SD-semantic-critical regions among the atrophic areas in addition to the FFG and temporal pole.

The present study aims to identify SD-semantic-critical regions by correlating semantic task performance with the gray matter index in the whole brain in 19 individuals with SD. The analyses further controlled for the influence of atrophy in multiple cerebral areas and potential confounding factors. We found that the left fusiform gyrus (lFFG) contributed to the semantic impairments of SD.

## Materials and Methods

### Participants

Patients with SD and healthy controls were recruited from Huashan Hospital in Shanghai. All participants were native Chinese speakers and provided written informed consent. This study was approved by the Institutional Review Board of the State Key Laboratory of Cognitive Neuroscience and Learning, Beijing Normal University.

#### SD Patients

Nineteen adults with SD participated in this study (12 males, all right-handed; mean age: 61.26 ± 8.63 years; formal education level: 11.53 ± 3.34 years). They had normal or corrected-to-normal hearing and vision, and no history of alcoholism, head trauma, or neurological or psychiatric illness. Their neuropsychological performance and predominant anterior temporal lobe atrophy met the diagnostic criteria for SD (Gorno-Tempini et al., 2011; see below for further description of these criteria). The mean interval between the behavioral and neuroimaging data collection was 41.47 ± 71.98 days (see Supplementary Table S1 for details).

#### Healthy Controls

Twenty healthy adults were selected as normal controls (8 males, all right-handed; mean age: 60.50 ± 3.93 years; formal education level: 10.45 ± 2.89 years). The subjects also had normal or corrected-to-normal hearing and vision, and no history of alcoholism, head trauma, or neurological or psychiatric illness. The same neuropsychological and neuroimaging data were collected from the control subjects as the SD patients. The mean interval between the behavioral and neuroimaging data collection was 46.65 ± 42.37 days (see Supplementary Table S2 for details).

There were no significant differences between the SD individuals and healthy controls in gender (*x*^2^ = 2.09, *p* = 0.20), age (*t* = 0.35, *p* = 0.72), education level (*t* = 1.08, *p* = 0.28), or the interval between the behavioral and imaging data collection (*t* = -0.28, *p* = 0.78).

### Behavioral Data Collection and Preprocessing

#### Data Collection

To confirm that our patients suffered from SD and to identify SD-semantic-critical regions, the SD patients and healthy controls underwent multiple behavioral assessments. SD is characterized by a progressive loss of semantic knowledge with evident anterior temporal lobe atrophy (Hodges et al., 1992; Hodges and Patterson, 2007). Gorno-Tempini et al. (2011) specified that individuals with SD exhibited behavioral deficits in confrontation naming, single-word comprehension, object knowledge (especially for low-frequency or low-familiarity objects), and surface dyslexia (or surface dysgraphia) with spared repetition and speech production (see **Table 1**). Therefore, we examined these profiles in our patients using the tests specified below. Probes of semantic processing ability (e.g., confrontation naming and single-word comprehension) are crucial for ascertaining semantic deficits and SD-semantic-critical regions; therefore, the participants were evaluated using multiple tasks that varied in stimulus input and output modalities (these tasks were used in our recent studies; e.g., Han et al., 2013a,b; Fang et al., 2015). Each task was tested in separate sessions in which the order of presentation was randomized but identical across subjects. The participants were tested individually in a quiet room. Each session lasted no more than 2 h. Rest breaks were allowed upon request.

**Table 1:** Demographic characteristics, behavioral performance (raw and corrected *t* scores) and cerebral gray matter volumes (GMVs) of SD patients and healthy control subjects.

		Healthy controls	SD patients	
		Raw score	Raw score	Corrected *t* score
**Demographic characteristics**				
Age (years)		60.50 (3.93)	61.26 (8.63)	
Gender (male:female)		8:12	12:7	
Education level (years)		10.45 (2.89)	11.53 (3.34)	
Handedness (right:left)		20:0	19:0	
Behavior-imaging data collection interval (days)		46.65 (42.37)	41.47 (71.98)	
**Behavioral performance**				
Confrontation naming	Oral picture naming (*n* = 140)	124.25 (7.95)	33.68 (21.07)	-10.26 (2.65)
	Oral sound naming (*n* = 36)	25.40 (4.06)	6.79 (4.38)	-4.31 (1.35)
Single-word comprehension	Picture associative matching (*n* = 70)	66.45 (2.39)	50.42 (7.87)	-5.36 (2.76)
	Word associative matching (*n* = 70)	67.15 (1.46)	49.16 (8.89)	-18.32 (10.10)
	Word-picture verification (*n* = 70)	67.25 (1.94)	40.11 (14.23)	-12.46 (6.87)
Object knowledge for low-frequency concepts	Naming to definition (*n* = 22)	18.35 (2.43)	4.59 (3.86)	-7.83 (3.07)
Surface dyslexia	Regularity effect of word reading aloud (the correct numbers on irregular words – those on regular words)	-0.40 (0.82)	-2.16 (2.24)	-2.45 (2.83)
	Regularization errors of word reading aloud (max = 12)	0.40 (0.75)	1.89 (1.37)	2.58 (2.23)
Repetition	Oral repetition (*n* = 12)	11.55 (0.94)	11.00 (1.37)	-0.44 (1.18)
Grammar processing	Percentage of reasonable sentences for cookie theft picture description (accuracy)	91% (13%)	87% (14%)	-0.35 (0.96)
Arithmetic ability	Number calculation (*n* = 7)	6.50 (0.69)	6.32 (1.06)	-0.24 (1.52)
General cognitive state	MMSE (max = 30)	27.95 (1.61)	20.47 (4.40)	-3.81 (2.21)
Visuospatial perception	REY-O copy (max = 36)	34.75 (1.77)	32.00 (4.38)	-1.62 (2.54)
Episodic memory	REY-O recall (max = 36)	16.05 (6.53)	8.94 (7.99)	-1.23 (1.16)
Executive function	STT (seconds)	91.35 (36.05)	121.00 (74.56)	0.87 (2.09)
**Cerebral gray matter volume**				
In the whole brain (cm^3^)		421 (28)	372 (39)	-1.62 (1.16)
In unilateral temporal pole (cm^3^)	Left temporal pole	5.09 (0.78)	1.35 (0.52)	-3.92 (0.66)
	Right temporal pole	6.62 (0.78)	2.90 (1.80)	-4.27 (1.95)

*The numbers in parentheses are the standard deviations*.

##### Confrontation naming

This characteristic was assessed using two tasks with different input modalities to ensure that the naming difficulty was not related to visual perception/recognition deficits. (**1) *Oral picture naming:*** This task contained 140 items, including 20 items from each of seven categories (animals, tools, common artifacts, fruits, and vegetables, large non-manipulable objects, faces, and actions). The participants were instructed to name each picture. (**2) *Oral sound naming:*** This task contained 36 items comprising the sounds of animals, tools, common artifacts, and other objects and events (e.g., thunder). The participants heard the target sound through earphones and were instructed to say what produced the sound (e.g., thunder).

##### Single-word comprehension

We designed three tasks with different types of input stimuli to ensure that comprehension disorders were apparent for various stimulus types. (**1) *Picture associative matching:*** This task contained 70 items, including 10 items from each category in the oral picture naming task described above. Each item contained three pictures in the same category arranged in an upright triangle. The participants were asked to identify which of the two bottom photographs (e.g., tadpole and lion) was semantically closer to the top one (e.g., frog). (**2) *Word associative matching:*** This task was identical to the picture associative matching task except that the pictures were replaced with corresponding written words. **(3) *Word-picture verification:*** This task contained 70 items, including 10 items from each category in the oral picture naming task described above. For each item, a photographed object (e.g., tiger) was presented in two separate blocks, once with the target object word (e.g., tiger) and once with a semantically related object word from the same category (e.g., leopard). The participants were asked to determine whether the object and the word were identical by pressing a “YES” or “NO” button on the screen. An item was scored as correct only if a correct response was provided for both blocks (i.e., the participant correctly accepted the target and rejected the incorrect choice).

##### Object knowledge for low-frequency concepts

The ***naming to definition task*** was used. This task contained 22 object items from the categories in the oral picture naming task described above. The written names of the items were infrequent (word frequency: 2 ± 0.9/million; Sun et al., 1997). For each item, the participants heard the definition of an object, which was adopted from an encyclopedia in most cases, and were told to say the name of the object.

##### Surface dyslexia

The ***word reading aloud task*** was used. We selected 24 Chinese semantic-phonetic compound characters. Each of the characters contained a semantic radical and a phonetic radical, which provide clues about the meaning and the pronunciation of the whole character, respectively (Weekes and Chen, 1999; Shu et al., 2003). For example, the compound character 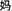/ma1/(mother) comprises the semantic radical 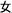/nv3/(female) and the phonetic radical 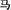/ma3/(horse). The compound characters consisted of 12 regular characters with sounds that are identical to those of their phonetic radicals and 12 irregular characters with sounds that differ from their phonetic radicals. The participants were asked to read the characters aloud and accurately. A patient was diagnosed with surface dyslexia if, relative to healthy controls, he or she had lower reading accuracy for irregular characters than regular characters or had a higher rate of regularization errors (Bi et al., 2007).

##### Repetition

The ***oral repetition task*** was adopted. The participants were asked to repeat what they heard (eight words, four sentences).

##### Grammar processing

We used the ***cookie theft picture description task*** from the Boston Diagnostic Aphasia Examination (BDAE; Goodglass and Kaplan, 1972). The participant’s grammar processing ability was defined as the percentage of reasonable sentences among all the sentences that he or she produced (Gordon, 2006).

##### General cognitive state

The Chinese version of the ***Mini-Mental State Examination*** (MMSE; Folstein et al., 1975) was used as a measure of the general cognitive state.

##### Arithmetic ability

We used a***calculation number task***. Seven calculation questions (two addition, two subtraction, two multiplication, and one division) were presented, and the subjects were instructed to provide the correct answers.

##### Episodic memory, visuospatial perception, and executive function

These abilities were investigated using the ***Rey-O Recall test*** (Rey, 1941; Osterrieth, 1944), the ***Rey-O Copy test*** (Rey, 1941; Osterrieth, 1944) and the shape trail test (STT; Zhao et al., 2013), respectively.

#### Data Preprocessing

##### Obtaining raw scores for each task

The first responses to the judgment tasks and the first complete responses to the oral production tasks (except for the cookie theft picture description task) were scored. The MMSE, Rey-O Recall test, Rey-O Copy test and STT were coded using their respective scoring standards. To ensure high rater-reliability of the tasks with scores based on the rater’s subjective judgments, two raters independently scored all such tasks for each SD patient. The scores of the two raters were significantly correlated for each task across patients (oral picture naming: *r* = 0.997, *p* < 10^-19^; oral sound naming: *r* = 0.99, *p* < 10^-15^; oral definition naming: *r* = 0.999, *p* < 10^-17^; oral word reading: *r* = 0.99, *p* < 10^-14^; *r* = 0.997, *p* < 10^-19^; oral repetition: *r* = 0.90, *p* < 10^-6^; MMSE: *r* = 0.996, *p* < 10^-18^).

##### Correcting the raw scores of each task

The patients’ raw scores may not have accurately reflected the degree of deficits, as the patient group showed considerable variation in demographic characteristics (e.g., age, gender, and education level; see Supplementary Table S1). To obtain an index that could more precisely measure the degree of deficits, we used the single case-to-controls method proposed by Crawford and Garthwaite (2006), in which the patients’ raw scores were corrected by considering the raw score distribution and demographic information of the 20 healthy subjects (see a detailed description for this method in Han et al., 2013a,b).

##### Computing the semantic score

The semantic performance of a patient was measured as a semantic composite score, which was computed using a principle component analysis (PCA) based on all six semantic tasks that varied in the degree of semantic involvement and input/output modalities, which included oral picture naming, sound naming, picture associative matching, word associative matching, word-picture verification, and naming to definition (low frequency). We entered the behavioral accuracies of all six tasks into the PCA program and employed varimax rotation, a plot of the eigenvalues (eigenvalues > 1) and a principal component extraction. The semantic PCA factor was defined as a component that had a high loading weight on all tasks in which semantic processing is highly relevant. The semantic PCA score was computed as the linear combination of the corrected task scores and factor score coefficients.

### Imaging Data Collection and Preprocessing

#### Data Collection

The SD patients and healthy control subjects were scanned using the same Siemens 3T scanner at Huashan Hospital in Shanghai. The 3D T1-weighted magnetization-prepared rapid gradient echo (MPRAGE) images (structural images) were acquired in the sagittal plane using the following parameters: repetition time = 2300 ms, echo time = 2.98 ms, flip angle = 9°, matrix size = 240 × 256, field of view = 240 mm × 256 mm, slice number = 192 slices, slice thickness = 1 mm, and voxel size = 1 mm × 1 mm × 1 mm.

#### Data Preprocessing

The structural images were first subjected to skull-strip processing using PANDA software^1^(Cui et al., 2013). The skull-stripped images were further resampled into 1.5 mm × 1.5 mm × 1.5 mm regions and segmented into different tissue types (i.e., gray matter, white matter, or cerebrospinal fluid), followed by spatial normalization to the MNI space using VBM8^2^ in SPM8^3^. Then, the gray matter volume (GMV) images were generated via affine transformation and non-linear warping and were smoothed using an 8-mm full-width at half-maximum Gaussian kernel.

### Identifying SD-Semantic-Critical Regions

Prior to identifying the SD-semantic-critical regions, we determined the SD-cortical-atrophic and SD-semantic-correlated regions.

#### SD-Cortical-Atrophic Regions

To locate the brain regions that clearly display atrophy in SD, we conducted a voxel-based morphometric analysis of the structural images in which an independent sample *t*-test was used to compare the GMVs of each voxel of the whole brain between SD patients and healthy control subjects. The voxels that survived at the significance of the AlphaSim-corrected *p* < 0.05 (single voxel *p* < 0.05, cluster size > 2885 voxels) threshold were used to create a binary atrophy mask.

We performed two complementary analyses in parallel, which included region-based and voxel-based analyses that were implemented on individual atrophic regions and individual atrophic voxels, respectively. The procedures for these two analyses were highly similar; therefore, we only describe the procedures for the region-based analysis (RBA) here. To identify the atrophic regions in the patients with SD, the atrophic mask obtained following the procedures described above was overlapped onto the automated anatomical labeling (AAL) atlas (Tzourio-Mazoyer et al., 2002), which parcellates the cerebral gray matter into 90 individual regions. An SD-cortical-atrophic region was defined as an AAL region with more than 50% of atrophic voxels (the number of voxels in the atrophy mask for the region/the total number of voxels in the region). Each patient was entered in the following analyses, regardless of his or her severity of atrophy in the SD-cortical-atrophic region. The atrophy value of a patient in an SD-cortical-atrophic region was the mean GMV of all voxels in the atrophy mask for the region.

#### SD-Semantic-Correlated Regions

To reveal the regions associated with semantic deficits in SD, the GMVs of each SD-cortical-atrophic region identified as described above were correlated with the semantic PCA scores across 19 SD patients. A region was considered as a SD-semantic-correlated region if it displayed a significant correlation (Bonferroni corrected *p* < 0.05).

#### SD-Semantic-Critical Regions

To find the critical regions of semantic impairments in SD, we separately correlated the GMVs of each SD-semantic-correlated region identified with the procedures described above using the patient’s semantic PCA scores after partialling out the GMVs of all other SD-semantic-correlated regions.

### Validating the Effects of an SD-Semantic-Critical Region

To examine whether the observed effects might be driven by other confounding variables, we again correlated the GMVs of the observed region with the semantic PCA scores across all SD patients, while controlling for the effects of other potential confounding variables. The potential confounding variables included (1) total GMV (measured by the total GMV of all voxels in the whole-brain gray matter mask), (2) overall cognitive state (the corrected *t* score of the MMSE test), (3) laterality of brain atrophy (a dichotomic variable: left- or right-hemispheric predominate atrophy was coded as 1 or 0, respectively), and (4) non-semantic control task performance (the corrected *t* scores on three minimal semantic processing tasks: episodic memory, oral word reading, and number calculation).

### Investigating the Semantic-Relevant Effects across Different Portions of an SD-Semantic-Critical Region

To explore whether the observed effects of an SD-semantic-critical region were driven by a given part of its atrophic region, we first evenly split the region (in MNI space) along the *y*-axis into five subregions. Then, the GMVs of each subregion were correlated with the semantic PCA scores across 19 SD subjects.

## Results

### Neuropsychological Profiles of the Participants

**Table 1** displays the background characteristics, behavioral performance (raw and corrected *t* scores) and cortical GMV of the SD patients and the healthy control subjects (see Supplementary Tables S1 and S2 for details). To evaluate whether a patient suffered from SD, we established the threshold value for his or her behavioral performance on each task and for the degree of atrophy in the temporal pole using raw scores (beyond two standard deviations from the average raw score of 20 healthy control subjects) or the corrected *t* scores (<-1.96 or >1.96). These two cutoff methods consistently revealed that the 19 patients exhibited the behavioral patterns and brain atrophy characteristics of SD, as proposed by Gorno-Tempini et al. (2011). Here, we present only the corrected *t* scores.

Our patient group exhibited profound deficits on the confrontation naming tasks (mean *t* scores < -4), single-word comprehension tasks (mean *t* scores < -5), and object knowledge task for infrequent objects (mean *t* score < -7). They also suffered from surface dyslexia with a clear regularity effect for reading words (mean *t* score < -2) and considerable regularization errors (mean *t* score > 2). Conversely, their repetition, grammar processing, arithmetic calculation, visuospatial perception, episodic memory, and executive function abilities were normal (mean *t* scores > -1.70). The semantic composite score was determined by performing a PCA across six semantic tasks. Only one component was extracted (eigenvalues >1), and this component accounted for 65% of the model variance with high loading values on each task (0.71–0.91). We thus labeled this component as the semantic processing component.

For brain atrophy, the patients exhibited clearly low GMVs in the left (mean *t* score < –3) and right (mean *t* score < –4) temporal poles. They presented with bilateral but asymmetric hemispheric atrophy (L > R: 13 patients; R > L: 6 patients).

### SD-Semantic-Critical Regions

#### SD-Cortical-Atrophic Regions

**FIGURE 1:**
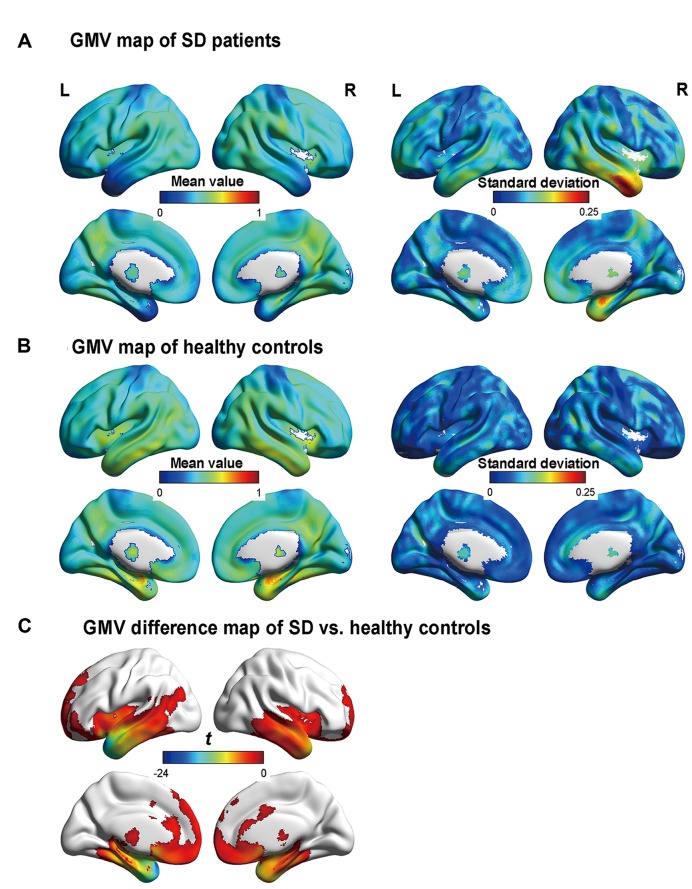
**Gray matter volume (GMV) maps of the participants. (A)** and **(B)** are the GMV maps of the SD patients and healthy controls, respectively. The value of each voxel is the mean value (i.e., the left column) or the standard deviation (i.e., the right column) of the GMV. **(C)** The regions with significant differences in GMV between the semantic dementia (SD) patients and healthy controls, with AlphaSim-corrected *p* < 0.05 (individual voxel: *p* < 0.05; cluster size > 2885 voxels). More negative values denote more severe atrophy.

**FIGURE 2:**
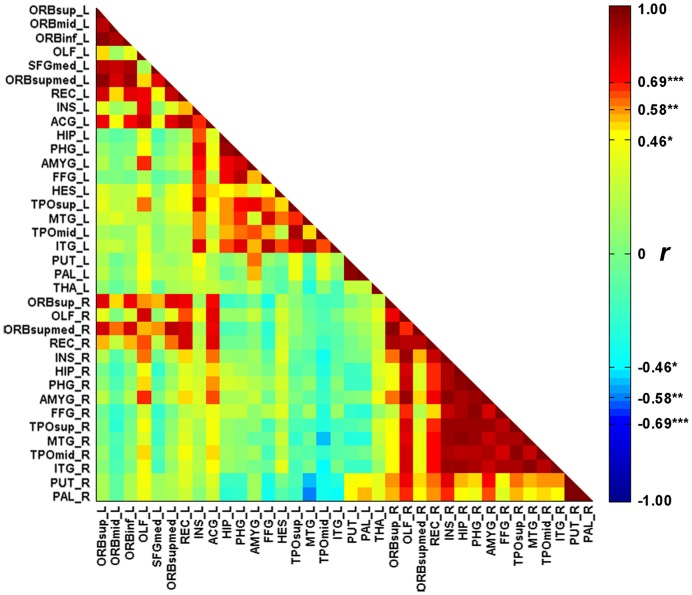
**The correlation matrix between the 36 cortical atrophic regions in SD patients.** The full names of abbreviations of the regions are given in **Table 2**. ^∗^*p* < 0.05, ^∗∗^*p* < 0.01, ^∗∗∗^*p* < 0.001.

**Figure 1** illustrates the GMVs of the individuals with SD and healthy controls. The maximal atrophy severity of SD was located in the left temporal pole (MNI coordinates: -54, 18, -28; **Figure 1C**). The atrophy mask covered 63 AAL regions (111,445 voxels). Among these regions, 36 (57%) met the criterion for a SD-cortical-atrophic region (atrophy percentage > 50%; **Table 2**). The GMVs of 220 pairs of atrophic regions showed significant correlations (*p*s < 0.05). These significant correlations mainly occurred within the frontal and temporal lobes, and between the frontal lobes bilaterally (**Figure 2**). The following region-based and voxel-based analyses were performed on 36 regions and 111,445 voxels, respectively.

**Table 2:** The characteristics of the 36 atrophic regions in SD patients and the correlation coefficients between the GMVs of each atrophic region and the semantic PCA scores.

Atrophic region	Abbr	Atrophy ratio in the whole region	Atrophy size (mm^3^)	Correlation coefficients
**Frontal lobe**				
Left superior frontal gyrus (orbital part)	ORBsup_L	0.67	5434	-0.12
Right superior frontal gyrus (orbital part)	ORBsup_R	0.64	5113	-0.29
Left middle frontal gyrus (orbital part)	ORBmid_L	0.60	4290	-0.13
Left inferior frontal gyrus (orbital part)	ORBinf_L	0.61	8043	-0.15
Left olfactory cortex	OLF_L	0.98	2238	0.17
Right olfactory cortex	OLF_R	0.98	2325	-0.01
Left superior frontal gyrus (medial orbital)	ORBsupmed_L	0.99	6058	-0.17
Right superior frontal gyrus (medial orbital)	ORBsupmed_R	0.91	6558	-0.30
Left rectus gyrus	REC_L	0.98	6591	0.00
Right rectus gyrus	REC_R	1.00	5984	-0.20
Left superior frontal gyrus (medial)	SFGmed_L	0.62	14435	-0.21
**Temporal lobe**				
Left amygdala	AMYG_L	1.00	1650	0.39
Right amygdala	AMYG_R	1.00	1961	0.05
Left hippocampus	HIP_L	0.99	7422	0.60^∗∗^
Right hippocampus	HIP_R	0.98	7526	0.07
Left parahippocampal gyrus (left parahippocampal gyrus)	PHG_L	1.00	7739	0.70^∗∗∗^
Right parahippocampal gyrus	PHG_R	0.94	8421	0.23
Left fusiform gyrus (lFFG)	FFG_L	0.75	13679	0.87^∗∗^
Right fusiform gyrus	FFG_R	0.61	12302	0.28
Left inferior temporal gyrus	ITG_L	0.96	24543	0.67^∗∗^
Right inferior temporal gyrus	ITG_R	0.75	21131	0.00
Left middle temporal gyrus	MTG_L	0.82	31877	0.64^∗∗^
Right middle temporal gyrus	MTG_R	0.54	19616	0.07
Left heschl gyrus	HES_L	0.65	1175	0.33
Left temporal pole: middle temporal gyrus	TPOmid_L	1.00	5987	0.31
Right temporal pole: middle temporal gyrus	TPOmid _R	1.00	9315	-0.15
Left temporal pole: superior temporal gyrus	TPOsup_L	0.97	9923	0.43
Right temporal pole: superior temporal gyrus	TPOsup_R	0.98	10493	-0.09
**Insula cortex**				
Left insula	INS_L	0.72	10986	0.49^∗^
Right insula	INS_R	0.81	11644	-0.02
**Cingulate cortex**				
Left anterior cingulate and paracingulate gyri	ACG_L	0.53	5933	0.20
**Basal ganglia**				
Left lenticular nucleus, pallidum	PAL_L	0.67	1468	-0.20
Right lenticular nucleus, pallidum	PAL_R	0.57	1218	-0.36
Left lenticular nucleus, putamen	PUT_L	0.8	6456	-0.07
Right lenticular nucleus, putamen	PUT_R	0.66	5633	-0.31
**Thalamus**				
Left thalamus	THA_L	0.52	4658	0.01

*^∗^p < 0.05, ^∗∗^p < 0.01, ^∗∗∗^p < 0.001.*

Note that the bilateral temporal poles had severe atrophy and GMV values on them reached a floor effect. Therefore, it was difficult to determine whether these two regions were SD-semantic-critical regions.

#### SD-Semantic-Correlated Regions

**FIGURE 3:**
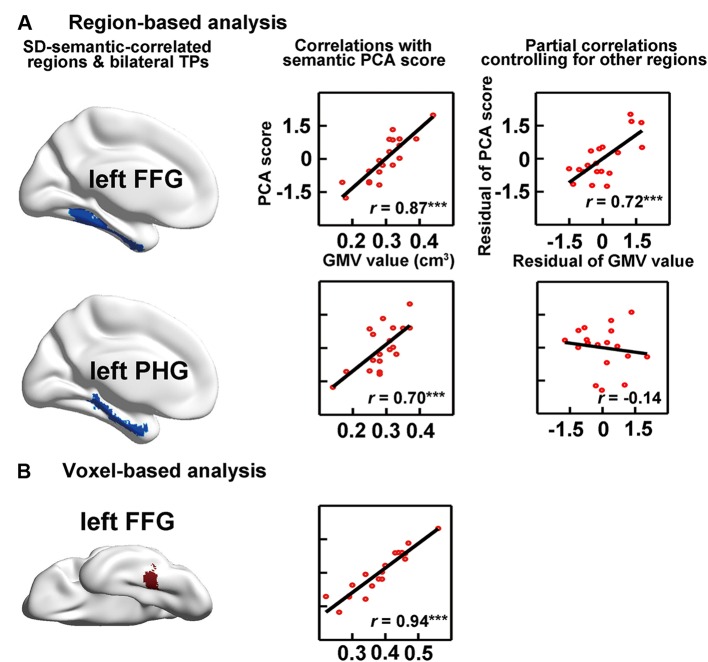
**Correlations between semantic performance and the GMVs on SD-semantic-correlated regions that were derived from region-based analysis (RBA; A) or voxel-based analysis (VBA; B).** The first column is the schematic of the SD-correlated regions in SD individuals. The middle column indicates the correlations between the GMVs of each region with the semantic PCA scores. The right column is the partial correlation between the GMVs of each region and the PCA scores, after controlling for the GMVs of other SD-correlated regions. ^∗∗∗^*p* < 0.001. FFG, fusiform gyrus; PHG, parahippocampal gyrus.

The RBA revealed two SD-semantic-correlated regions whose GMV values were significantly positively correlated with the semantic PCA scores [lFFG: *r* = 0.87, *p* < 0.00002 and left parahippocampal gyrus (lPHG): *r* = 0.70, *p* < 0.0009, Bonferroni corrected *p* < 0.05; see **Table 2**, **Figure 3A**]. The voxel-based analysis (VBA) identified only one cluster in which the GMVs of each voxel were significantly correlated with the semantic PCA scores (cluster size: 383 voxels; mean *r* = 0.94, Bonferroni corrected *p* < 0.05, peak point: *x* = -34, *y* = -48, *z* = -16). The cluster was located in the lFFG (see **Figure 3B**).

#### SD-Semantic-Critical Regions

We investigated the effects of the GMVs of each SD-semantic-correlated region with the semantic PCA scores after controlling for the GMVs of the other SD-semantic-correlated region. The RBA revealed that the effect of the lFFG remained significant (partial *r* = 0.72, *p* < 0.0008), but the effect of the lPHG was not significant (partial *r* = -0.14, *p* = 0.59; **Figure 3A**). In brief, the analysis suggested that the lFFG might be a SD-semantic-critical region.

### Validating the Effects of an SD-Semantic-Critical Region

To further confirm the SD-semantic-critical effects of the lFFG, we factored out the influence of potential confounding variables. The results revealed that the GMV values of the lFFG still significantly correlated with the semantic PCA scores after partialling out the influence of the total GMV (RBA: partial *r* = 0.84, *p* < 0.00002; VBA: partial *r* = 0.92, *p* < 10^-7^), general cognitive processing (RBA: partial *r* = 0.80, *p* < 0.00007; VBA: partial *r* = 0.91, *p* < 10^-6^), laterality of brain atrophy (RBA: *r* = 0.89, *p* < 10^-6^; VBA: partial *r* = 0.93, *p* < 10^-7^), performance on the non-semantic control tasks (RBA: partial *r* = 0.87, *p* < 0.00002; VBA: partial *r* = 0.94, *p* < 10^-6^), and all of the above potentially confounding variables (RBA: partial *r* = 0.81, *p* < 0.0009; VBA: partial *r* = 0.92, *p* < 0.00001). The above results further showed that these factors could not fully account for the effects of the lFFG.

Note that five of the six semantic tasks from which the PCA semantic scores were extracted were verbal tasks, and only one of them was non-verbal task (i.e., picture associative matching). Thus, it is possible that the extent to which the higher correlations with the left FFG than the right FFG for semantic performance resulted from the involvement of verbal processing for most of the tasks. To test this possibility, we compared the effects of non-verbal semantic task on bilateral fusiform gyri. We found that GMVs of both regions were significantly correlated with corrected *t* scores of picture associative matching task (RBA: left FFG: *r* = 0.58, *p* < 0.01; right FFG: *r* = 0.48, *p* < 0.05; VBA: left FFG: *r* = 0.62, *p* < 0.005; right FFG: *r* = 0.50, *p* < 0.03). There was no significant difference between the two correlation values (RBA: *t* = 0.42, *p* = 0.68; VBA: *t* = 0.66, *p* = 0.52). The findings suggest that bilateral fusiform gyri may both attribute to non-verbal semantics.

In addition, four of our six semantic tasks involved visual stimuli input. A relevant question is whether the observed effects of the lFFG were driven by visual but not semantic processing. To address this issue, we again performed a correlation analysis with the GMVs of the lFFG using the corrected *t* scores from the non-visual task (oral sound naming and naming to definition). The correlations were still significant (oral sound naming: RBA: *r* = 0.56, *p* < 0.02, VBA: *r* = 0.75, *p* < 0.0003; naming to definition: RBA: *r* = 0.68, *p* < 0.003, VBA: *r* = 0.75, *p* < 0.0005). This result shows that the atrophy of the lFFG also led to disruptions of non-visual semantic processing. Therefore, the lFFG should contribute to semantic deficits of SD.

### The Semantic-Relevant Effects across Different Portions of an SD-Semantic-Critical Region

**FIGURE 4:**
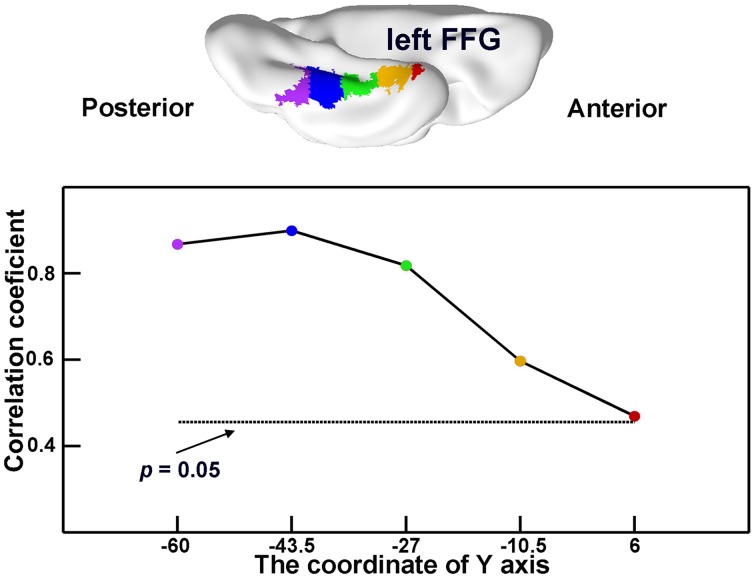
**The semantic-relevant effects across different portions of the left fusiform gyrus (lFFG).** The lFFG was first evenly split into five subregions along the *y*-axis **(top)**. Then, the GMVs of each subregion were correlated with the semantic composite scores across SD subjects **(bottom)**. The horizontal dotted line shows the threshold for significance for the correlation coefficients (*p* < 0.05).

In the atrophic mask of the lFFG, the GMVs of each subregion significantly correlated with semantic scores in SD subjects (*r* = 0.47 to 0.90; all *p*s < 0.05; **Figure 4**). This demonstrates that each atrophic portion of the lFFG along the anterior–posterior axis may play an important role of semantic deterioration in SD.

## Discussion

The aim of the present study is to address the neuroanatomical basis for semantic deficits in SD. Using behavioral and structural brain data from 19 individuals with SD, we found 36 regions with gray matter atrophy. These regions were primarily distributed bilaterally in the temporal, ventral frontal and insular cortices. Among these atrophic regions, atrophy of the lFFG and lPHG were associated with semantic impairments in SD, as the reduction of their GMVs significantly correlated to the severity of semantic deficits. The lFFG was further identified as a critical region of semantic deficits in SD, as its volume reduction remained significant correlation with the severity of semantic impairments in SD after partialling out the influence of the lPHG. The relationship between the lFFG and the decline in semantic performance in patients with SD remained significant even after controlling for a range of potential confounding factors (i.e., total GMV, overall cognitive state, laterality of brain atrophy, and non-semantic control task scores). Moreover, the observed effects of the atrophic lFFG cannot be simply deduced as deficits of visual processing. Note that the temporal poles had severe atrophy so that it is difficult to find temporal poles’ effect due to a floor effect. In sum, the current study demonstrated that the lFFG dedicated to semantic deficits of SD. The following section addresses the implications associated with these brain regions in SD.

### The Fusiform Gyrus

Although researchers have discovered many atrophic regions in individuals with SD, which of these atrophic regions actually lead to the semantic deficits observed in SD is unknown (Chan et al., 2001; Desgranges et al., 2007, but see Mion et al., 2010). The present study reveals that the lFFG plays a critical role in the semantic deficits observed in SD. In fact, Mion et al. (2010) suggested that dysfunction of this region could cause a loss of semantic functions in SD. Our results further expand the findings of Mion et al. (2010) in the several ways. First, the effects of the lFFG as an SD-semantic-critical region were previously established through functional measures (Mion et al., 2010) and by anatomical measures in the present study. Second, previous findings distinguished between the two regions most related to semantic functions, the FFG and the temporal pole (Mion et al., 2010), whereas the present study distinguished these functions from atrophic regions throughout the brain. Third, the effect of lFFG in SD were identified for verbal semantic processing in the previous study (Mion et al., 2010), whereas non-verbal performance was also related to lFFG in the present study. Finally, the present study revealed similar roles of the anterior-to-posterior portions of the lFFG in the semantic deficits of SD.

In fact, the lFFG, and especially its anterior part, has been considered an amodal region for semantic representation (Binney et al., 2010; Lambon Ralph, 2014). Given that the lFFG is adjacent to multiple modality-specific regions, such as auditory, visual, olfactory, and emotional systems (Rice et al., 2015), the lFFG may be responsible for amodal semantic representation of single objects (Binney et al., 2012). The lFFG can also synthesize verbal and non-verbal information from the left lateral temporal lobe and the right temporal lobe, respectively. Considerable evidence from prior studies has shown the important functions of the lFFG for semantic processing, including the findings from meta-analyses (Binder et al., 2009; Visser et al., 2010), cortical stimulation (Shimotake et al., 2014), PET or distortion corrected fMRI (Visser and Lambon Ralph, 2011; Visser et al., 2012) and neuropsychological studies (Wilson et al., 2015; Wright et al., 2015).

### The Temporal Pole

The temporal pole has been considered an SD-semantic-critical region because this region shows the earliest and most severe atrophy in SD (Lambon Ralph et al., 2009; Lambon Ralph et al., 2010). However, the findings of Mion et al. (2010) and our study regarding the effects of the temporal pole on the semantic deficits in SD did not achieve significance after the influences of other atrophic regions were considered. These negative results regarding the involvement of the temporal pole could be interpreted according to three possibilities. First, the temporal pole is associated with the semantic deficits observed in SD (Patterson et al., 2007), but the atrophy of this region demonstrated a floor effect in our study. Second, different regions of the temporal cortex process distinct types of semantic information: the FFG contributes to the concept of concrete objects or superordinate conceptual knowledge (Clarke and Tyler, 2015), whereas the temporal pole contributes to abstract concepts (Binder et al., 2005; Jefferies et al., 2009), unique entities (Pobric et al., 2007; Clarke and Tyler, 2014) or social information (Zahn et al., 2007; Simmons et al., 2009). Because our study only assessed the semantic processing of concrete objects in subjects with SD, we failed to confirm a role for the temporal pole in semantic processing. Finally, the temporal pole is not associated with SD-related semantic deficits, and the previously reported effects of this region were driven by other atrophic regions.

### Limitations

Our patients suffered from severe SD, and the GMVs of the temporal poles showed a floor effect that did not allow us to determine whether these regions were SD-semantic-critical regions. To resolve this problem, future studies should include a sample of SD patients with mild atrophy. Similarly, some regions unaffected by SD showed ceiling effects for neural atrophy. The roles of these regions in semantic processing were also not explored in our study. Therefore, other approaches, such as transcranial magnetic stimulation or functional MRI, should be used to address such issues.

## Conclusion

The lFFG contributes to the semantic impairments of SD, and the deterioration of this brain region causes semantic deficits in SD. These results highlight a critical role of the lFFG in the semantic deficits observed in SD. However, the GMV values of the temporal pole regions in our patients showed floor effects, which prevented us from excluding the possibility that these regions were SD-semantic-critical regions.

## Author Contributions

QG, YB, and ZH designed research. KC, QY, NL, and YL performed research. JD, KC, YF, and YC analyzed data. JD, KC, QG, YB, and ZH wrote the paper.

## Conflict of Interest Statement

The authors declare that the research was conducted in the absence of any commercial or financial relationships that could be construed as a potential conflict of interest.

## Footnotes

1 http://www.nitrc.org/projects/panda/

2 http://dbm.neuro.uni-jena.de/vbm8/

3 http://www.fil.ion.ucl.ac.uk/spm/
