# Supplementary material for: The Left Fusiform Gyrus is a Critical Region Contributing to the Core Behavioral Profile of Semantic Dementia
Source: Front Hum Neurosci. 2016 May 19;10:215. doi: 10.3389/fnhum.2016.00215 (PMC4871861; doi:10.3389/fnhum.2016.00215)
Supplement: Supplementary file 1 [file Table_1.DOCX]

**Supplementary Table 1.** The demographic characteristics, raw scores (corrected *t* scores) for the neuropsychological tasks and cerebral gray matter volumes for the SD patients.

| Patient | 1 | 2 | 3 | 4 | 5 | 6 | 7 | 8 | 9 | 10 | 11 | 12 | 13 | 14 | 15 | 16 | 17 | 18 | 19 |
| --- | --- | --- | --- | --- | --- | --- | --- | --- | --- | --- | --- | --- | --- | --- | --- | --- | --- | --- | --- |
| **Demographic characteristics** |  |  |  |  |  |  |  |  |  |  |  |  |  |  |  |  |  |  |  |
| Age (years) | 46 | 48 | 49 | 52 | 52 | 57 | 59 | 62 | 62 | 63 | 63 | 65 | 65 | 66 | 68 | 68 | 69 | 75 | 75 |
| Gender (male:female) | M | M | M | F | F | M | M | M | F | M | F | F | M | F | M | M | M | F | M |
| Education level (years) | 9 | 9 | 15 | 7 | 12 | 16 | 11 | 12 | 15 | 9 | 9 | 12 | 12 | 14 | 16 | 12 | 4 | 9 | 16 |
| Handedness (right:left) | R | R | R | R | R | R | R | R | R | R | R | R | R | R | R | R | R | R | R |
| Behavior-imaging data collection interval (days) | 0 | 10 | 16 | 3 | 0 | 15 | 12 | 63 | 0 | 0 | 60 | 14 | 248 | 0 | 8 | 12 | 213 | 13 | 101 |
| **Behavioral performance** |  |  |  |  |  |  |  |  |  |  |  |  |  |  |  |  |  |  |  |
| *Confrontation naming* |  |  |  |  |  |  |  |  |  |  |  |  |  |  |  |  |  |  |  |
| Oral picture naming (*n* = 140) | 44 (-9.00) | 34 (-9.95) | 34 (-11.34) | 47 (-10.30) | 19 (-13.53) | 44 (-10.13) | 14 (-12.25) | 52 (-8.13) | 17 (-12.10) | 85 (-3.95) | 9 (-13.48) | 17 (-11.97) | 14 (-12.27) | 35 (-9.63) | 15 (-12.97) | 29 (-10.40) | 50 (-6.49) | 11 (-11.41) | 70 (-5.69) |
| Oral sound naming (*n* = 36) | 17 (-1.99) | 9 (-3.54) | 8 (-4.92) | 6 (-5.37) | 6 (-5.86) | 10 (-4.33) | 6 (-4.14) | 5 (-4.44) | 3 (-5.92) | 12 (-2.24) | 3 (-5.42) | 0 (-6.17) | 3 (-4.77) | 12 (-3.46) | 7 (-4.49) | 3 (-4.63) | 12 (-1.13) | 1 (-4.81) | 6 (-4.31) |
| *Single-word comprehension* |  |  |  |  |  |  |  |  |  |  |  |  |  |  |  |  |  |  |  |
| Picture associative matching (*n* = 70) | 56 (-2.70) | 47 (-5.40) | 61 (-1.70) | 52 (-5.31) | 43 (-8.51) | 62 (-1.55) | 47 (-6.07) | 46 (-6.65) | 41 (-8.76) | 55 (-3.37) | 39 (-9.87) | 50 (-5.84) | 37 (-9.77) | 64 (-1.17) | 55 (-4.20) | 42 (-8.19) | 56 (-2.69) | 52 (-5.03) | 53 (-5.02) |
| Word associative matching (*n* = 70) | 47 (-21.38) | 48 (-20.21) | 47 (-23.81) | 54 (-17.71) | 33 (-40.54) | 64 (-4.73) | 41 (-26.29) | 59 (-7.78) | 49 (-18.85) | 62 (-3.87) | 36 (-33.57) | 44 (-23.41) | 42 (-24.71) | 59 (-7.38) | 48 (-19.27) | 51 (-14.61) | 46 (-16.48) | 42 (-22.00) | 62 (-1.58) |
| Word-picture verification (*n* = 70) | 41 (-11.98) | 38 (-13.16) | 40 (-13.67) | 45 (-12.54) | NaN | 64 (-2.02) | 31 (-16.42) | 52 (-6.87) | 29 (-17.49) | 62 (-1.98) | 9 (-28.53) | 22 (-20.92) | 26 (-18.80) | 56 (-4.65) | 37 (-14.28) | 36 (-13.92) | 48 (-7.06) | 34 (-13.94) | 52 (-6.04) |
| *Object knowledge for low-frequency concepts* |  |  |  |  |  |  |  |  |  |  |  |  |  |  |  |  |  |  |  |
| Naming to definition (*n* = 22) | 6 (-3.57) | 6 (-3.83) | 4 (-7.66) | 6 (-4.90) | NaN | 7 (-7.83) | 1 (-8.82) | 9 (-5.56) | 0 (-11.71) | 13 (-2.30) | 0 (-10.32) | 1 (-10.72) | 0 (-10.84) | 6 (-8.61) | 1 (-13.02) | 5 (-8.60) | 3 (-5.93) | NaN | 10 (-8.86) |
| *Surface dyslexia* |  |  |  |  |  |  |  |  |  |  |  |  |  |  |  |  |  |  |  |
| Regularity effect of word reading aloud (the correct numbers on irregular words - those on regular words) | -2 (1.19) | -3 (2.34) | -3 (3.82) | -1 (-0.34) | -1 (0.76) | -2 (3.22) | -3 (3.28) | 1 (-1.23) | -2 (2.81) | 0 (-0.56) | -4 (4.39) | -5 (6.12) | 0 (0.08) | 0 (0.31) | -6 (8.99) | -2 (2.68) | -4 (3.16) | -6 (6.99) | 2 (-1.37) |
| Regularization errors of word reading aloud (max = 12) | 3 (1.56) | 3 (1.76) | 4 (5.29) | 2 (0.67) | 0 (-0.94) | 1 (2.10) | 4 (5.06) | 0 (-0.16) | 2 (3.99) | 0 (-0.92) | 2 (2.49) | 2 (3.51) | 2 (3.17) | 2 (4.09) | 4 (8.24) | 1 (1.99) | 3 (2.39) | 1 (2.09) | 0 (2.60) |
| *Repetition* |  |  |  |  |  |  |  |  |  |  |  |  |  |  |  |  |  |  |  |
| Oral repetition (*n* = 12) | 12 (0.81) | 12 (0.78) | 10 (-0.98) | 12 (0.56) | 10 (-1.41) | 12 (0.51) | 7 (-3.49) | 12 (0.52) | 12 (0.07) | 12 (0.56) | 9 (-2.44) | 11 (-0.75) | 11 (-0.39) | 12 (0.02) | 12 (0.30) | 12 (0.41) | 10 (-0.96) | 10 (-1.70) | 11 (-0.76) |
| *Grammar processing* |  |  |  |  |  |  |  |  |  |  |  |  |  |  |  |  |  |  |  |
| Percentage of reasonable sentences for cookie theft picture description (accuracy) | 100% (0.39) | 100% (0.38) | 75% (-1.00) | 100% (0.84) | 100% (0.89) | 83% (-0.62) | 67% (-1.67) | 80% (-0.93) | 100% (0.74) | 100% (0.22) | 80% (-0.61) | 100% (0.68) | 100% (0.25) | 80% (-0.53) | 63% (-2.16) | 100% (0.21) | 86% (-0.72) | 75% (-1.05) | 67% (-2.01) |
| *Arithmetic ability* |  |  |  |  |  |  |  |  |  |  |  |  |  |  |  |  |  |  |  |
| Number calculation (*n* = 7) | 7 (0.97) | 6 (-0.11) | 7 (0.37) | 7 (0.87) | 4 (-3.77) | 7 (0.29) | 5 (-1.52) | 7 (0.79) | 6 (-1.40) | 7 (1.11) | 7 (0.60) | 5 (-2.31) | 7 (0.81) | 7 (-0.04) | 7 (0.34) | 7 (0.83) | 7 (1.63) | 4 (-3.13) | 6 (-0.98) |
| *General cognitive state* |  |  |  |  |  |  |  |  |  |  |  |  |  |  |  |  |  |  |  |
| MMSE (max = 30) | 22 (-2.75) | 20 (-3.64) | 23 (-2.89) | 21 (-3.82) | 17 (-6.08) | 24 (-2.46) | 13 (-7.31) | 22 (-3.04) | 22 (-3.18) | 24 (-1.77) | 13 (-7.84) | 16 (-6.10) | 25 (-1.51) | 26 (-1.08) | 19 (-5.04) | 21 (-3.52) | 20 (-3.12) | 13 (-7.20) | 28 (-0.07) |
| *Visuospatial perception* |  |  |  |  |  |  |  |  |  |  |  |  |  |  |  |  |  |  |  |
| REY-O copy (max = 36) | 35 (1.25) | 35 (1.18) | 35 (-0.06) | 29 (-2.36) | 31 (-2.29) | 35 (-0.69) | 36 (0.88) | 31 (-2.15) | 22 (-8.04) | 35 (0.60) | 31 (-2.13) | 35 (-0.63) | 24 (-6.12) | 36 (-0.56) | 36 (-0.69) | 30 (-3.04) | 33 (0.35) | 26 (-5.30) | 36 (-1.06) |
| *Episodic memory* |  |  |  |  |  |  |  |  |  |  |  |  |  |  |  |  |  |  |  |
| REY-O recall (max = 36) | 10 (-0.88) | 4 (-1.63) | 9 (-2.10) | 10 (-0.47) | 4 (-2.31) | 31 (1.12) | 6 (-1.62) | 4 (-2.07) | 0 (-3.03) | 12 (-0.34) | 13 (-0.10) | 0 (-2.52) | 0 (-2.62) | 22 (0.41) | 8 (-2.21) | 8 (-1.38) | 3 (-0.60) | 13 (0.16) | 14 (-1.12) |
| *Executive function* |  |  |  |  |  |  |  |  |  |  |  |  |  |  |  |  |  |  |  |
| STT (seconds) | 83 (1.53) | 58 (0.66) | 80 (1.85) | 173 (3.46) | NaN | 89 (1.18) | 54 (-0.67) | 101 (0.36) | NaN | 91 (-0.37) | 12 (-3.07) | NaN | 201 (2.85) | 243 (3.84) | 192 (2.77) | 122 (0.13) | 95 (-1.49) | 278 (3.15) | 64 (-2.28) |
| **Cerebral gray matter volume** |  |  |  |  |  |  |  |  |  |  |  |  |  |  |  |  |  |  |  |
| *In the whole brain (cm^3^)* | 408 (-0.75) | 357 (-2.13) | 348 (-2.88) | 374 (-1.36) | 405 (-0.75) | 417 (-0.80) | 416 (-0.40) | 348 (-2.41) | 289 (-4.21) | 397 (-0.69) | 329 (-2.64) | 300 (-3.63) | 427 (-0.01) | 394 (-0.84) | 387 (-1.44) | 372 (-1.56) | 394 (-0.28) | 340 (-1.87) | 361 (-2.04) |
| *In unilateral temporal pole (cm^3^)* |  |  |  |  |  |  |  |  |  |  |  |  |  |  |  |  |  |  |  |
| Left temporal pole | 1.21 (-4.39) | 1.80 (-3.82) | 1.15 (-4.55) | 2.02 (-3.94) | 1.24 (-4.34) | 1.70 (-3.81) | 1.10 (-4.35) | 1.65 (-3.74) | 0.41 (-4.32) | 1.23 (-4.11) | 0.43 (-4.94) | 1.97 (-2.84) | 1.37 (-3.95) | 1.40 (-3.19) | 1.02 (-4.24) | 0.64 (-4.64) | 1.22 (-3.92) | 1.68 (-2.87) | 2.40 (-2.43) |
| Right temporal pole | 2.58 (-4.99) | 1.14 (-6.38) | 5.47 (-2.29) | 2.14 (-5.75) | 4.12 (-3.13) | 5.75 (-1.68) | 3.77 (-3.60) | 0.33 (-7.40) | 1.58 (-5.22) | 1.79 (-5.46) | 2.93 (-3.95) | 1.46 (-5.35) | 4.72 (-2.36) | 5.81 (-0.33) | 5.30 (-1.68) | 1.51 (-5.89) | 2.40 (-4.24) | 1.64 (-4.56) | 0.70 (-6.91) |

NaN means that the patients did not finish the tasks for any reasons. MMSE= Mini-Mental State Examination, REY-O: Rey-Osterrieth Complex Figure Test, STT = shape trail test.

**Supplementary Table 2.** Demographic characteristics, raw scores for the neuropsychological tasks and cerebral gray matter volumes for the healthy subjects.

| Subject | 1 | 2 | 3 | 4 | 5 | 6 | 7 | 8 | 9 | 10 | 11 | 12 | 13 | 14 | 15 | 16 | 17 | 18 | 19 | 20 |
| --- | --- | --- | --- | --- | --- | --- | --- | --- | --- | --- | --- | --- | --- | --- | --- | --- | --- | --- | --- | --- |
| **Demographic characteristics** |  |  |  |  |  |  |  |  |  |  |  |  |  |  |  |  |  |  |  |  |
| Age (years) | 51 | 57 | 57 | 58 | 58 | 59 | 59 | 59 | 59 | 60 | 60 | 60 | 61 | 62 | 62 | 63 | 64 | 66 | 66 | 69 |
| Gender (male:female) | m | f | m | f | f | f | f | f | m | f | f | f | f | f | m | m | m | f | m | m |
| Education level (years) | 16 | 9 | 12 | 9 | 9 | 9 | 9 | 12 | 12 | 2 | 12 | 11 | 9 | 12 | 12 | 9 | 15 | 9 | 12 | 9 |
| Handedness (right:left) | r | r | r | r | r | r | r | r | r | r | r | r | r | r | r | r | r | r | r | r |
| Behavior-imaging data collection interval (days) | 8 | 34 | 136 | 5 | 0 | 93 | 7 | 38 | 28 | 8 | 64 | 37 | 75 | 1 | 150 | 69 | 62 | 43 | 33 | 42 |
| **Behavioral performance** |  |  |  |  |  |  |  |  |  |  |  |  |  |  |  |  |  |  |  |  |
| *Confrontation naming* |  |  |  |  |  |  |  |  |  |  |  |  |  |  |  |  |  |  |  |  |
| Oral picture naming (*n* = 140) | 138 | 116 | 118 | 131 | 123 | 112 | 126 | 136 | 130 | 127 | 124 | 120 | 131 | 129 | 128 | 123 | 132 | 111 | 112 | 118 |
| Oral sound naming (*n* = 36) | 32 | 27 | 26 | 26 | 26 | 19 | 31 | 32 | 17 | 23 | 29 | 24 | 27 | 25 | 22 | 26 | 29 | 23 | 23 | 21 |
| *Single-word comprehension* |  |  |  |  |  |  |  |  |  |  |  |  |  |  |  |  |  |  |  |  |
| Picture associative matching (*n* = 70) | 67 | 64 | 64 | 66 | 69 | 62 | 68 | 69 | 68 | 67 | 69 | 67 | 65 | 68 | 66 | 67 | 68 | 67 | 60 | 68 |
| Word associative matching (*n* = 70) | 70 | 68 | 67 | 67 | 68 | 67 | 67 | 69 | 68 | 67 | 67 | 68 | 68 | 68 | 67 | 67 | 67 | 65 | 64 | 64 |
| Word-picture verification (*n* = 70) | 70 | 67 | 67 | 69 | 68 | 63 | 68 | 70 | 68 | 67 | 68 | 67 | 68 | 68 | 70 | 67 | 64 | 66 | 64 | 66 |
| *Object knowledge for low-frequency concepts* |  |  |  |  |  |  |  |  |  |  |  |  |  |  |  |  |  |  |  |  |
| Naming to definition (*n* = 22) | 19 | 15 | 17 | 17 | 18 | 16 | 18 | 20 | 22 | 11 | 20 | 21 | 18 | 19 | 19 | 20 | 21 | 19 | 18 | 19 |
| *Surface dyslexia* |  |  |  |  |  |  |  |  |  |  |  |  |  |  |  |  |  |  |  |  |
| Regularity effect of word reading aloud (the correct numbers on irregular words - those on regular words) | 0 | -1 | 0 | -1 | 1 | -1 | 0 | 0 | 0 | -3 | -1 | -1 | 0 | 0 | 0 | 0 | 0 | -1 | 0 | 0 |
| Regularization errors of word reading aloud (max = 12) | 0 | 1 | 1 | 1 | 0 | 0 | 0 | 0 | 0 | 3 | 1 | 0 | 1 | 0 | 0 | 0 | 0 | 0 | 0 | 0 |
| *Repetition* |  |  |  |  |  |  |  |  |  |  |  |  |  |  |  |  |  |  |  |  |
| Oral repetition (*n* = 12) | 12 | 12 | 12 | 11 | 11 | 12 | 12 | 12 | 8 | 12 | 12 | 11 | 11 | 12 | 12 | 12 | 12 | 12 | 12 | 11 |
| *Grammar processing* |  |  |  |  |  |  |  |  |  |  |  |  |  |  |  |  |  |  |  |  |
| Percentage of reasonable sentences for cookie theft picture description (accuracy) | 86% | 80% | 100% | 83% | 100% | 50% | 78% | 100% | 100% | 100% | 100% | 100% | 100% | 88% | 100% | 100% | 100% | 86% | 75% | 100% |
| *Arithmetic ability* |  |  |  |  |  |  |  |  |  |  |  |  |  |  |  |  |  |  |  |  |
| Number calculation (*n* = 7) | 7 | 6 | 6 | 7 | 5 | 7 | 6 | 7 | 7 | 6 | 7 | 7 | 7 | 7 | 6 | 7 | 6 | 7 | 7 | 5 |
| *General cognitive state* |  |  |  |  |  |  |  |  |  |  |  |  |  |  |  |  |  |  |  |  |
| MMSE (max = 30) | 30 | 25 | 28 | 30 | 25 | 29 | 29 | 30 | 28 | 27 | 26 | 30 | 27 | 28 | 29 | 27 | 26 | 28 | 29 | 28 |
| *Visuospatial perception* |  |  |  |  |  |  |  |  |  |  |  |  |  |  |  |  |  |  |  |  |
| REY-O copy (max = 36) | 36 | 34 | 36 | 35 | 31 | 36 | 36 | 35 | 31 | 31 | 36 | 35 | 36 | 36 | 36 | 35 | 36 | 34 | 36 | 34 |
| *Episodic memory* |  |  |  |  |  |  |  |  |  |  |  |  |  |  |  |  |  |  |  |  |
| REY-O recall (max = 36) | 26 | 25 | 20 | 9 | 13 | 7 | 9 | 24 | 14 | 11 | 23 | 13 | 11 | 21 | 21 | 7 | 20 | 8 | 17 | 22 |
| *Executive function* |  |  |  |  |  |  |  |  |  |  |  |  |  |  |  |  |  |  |  |  |
| STT (seconds) | 34 | 77 | 39 | 114 | 49 | 79 | 73 | 104 | 114 | 132 | 70 | 83 | 98 | 101 | 145 | 72 | 59 | 166 | 76 | 142 |
| **Cerebral gray matter volume** |  |  |  |  |  |  |  |  |  |  |  |  |  |  |  |  |  |  |  |  |
| *In the whole brain (cm^3^)* | 427 | 401 | 423 | 416 | 427 | 418 | 471 | 422 | 468 | 397 | 416 | 425 | 361 | 429 | 406 | 427 | 430 | 396 | 478 | 383 |
| *In unilateral temporal pole (cm^3^)* |  |  |  |  |  |  |  |  |  |  |  |  |  |  |  |  |  |  |  |  |
| Left temporal pole | 5.69 | 4.75 | 5.59 | 5.11 | 5.87 | 4.48 | 6.26 | 5.28 | 5.38 | 4.92 | 5.22 | 5.21 | 3.71 | 4.30 | 4.57 | 4.97 | 3.48 | 4.70 | 6.60 | 5.81 |
| Right temporal pole | 7.51 | 5.70 | 7.22 | 6.82 | 6.86 | 5.24 | 8.15 | 7.05 | 7.13 | 6.44 | 6.46 | 6.26 | 5.33 | 6.47 | 6.24 | 6.83 | 6.27 | 5.72 | 7.94 | 6.67 |

MMSE= Mini-Mental State Examination, REY-O: Rey-Osterrieth Complex Figure Test, STT = shape trail test.
